# Examining noncommunicable diseases using satellite imagery: a systematic literature review

**DOI:** 10.1186/s12889-024-20316-z

**Published:** 2024-10-10

**Authors:** Elizabeth J. Folkmann, M. Courtney Hughes, Uzma Amzad Khan, Mahdi Vaezi

**Affiliations:** 1 Shirley Ryan AbilityLab, 355 East Erie Street, Chicago, IL 60611 USA; 2 School of Health Studies, College of Health and Human Sciences, Northern Illinois University, 209 Wirtz Hall, DeKalb, IL 60115 USA; 3 College of Business, Northern Illinois University, 328 Barsema Hall, DeKalb, IL USA; 4 College of Engineering and Engineering Technology, Northern Illinois University, 590 Garden Road, DeKalb, IL USA

**Keywords:** Geospatial Epidemiology, Noncommunicable disease, Chronic disease, Population Health, Satellite Imagery, Systematic review, Heart Disease, Cancer, Diabetes, Asthma

## Abstract

**Introduction:**

Noncommunicable diseases (NCDs) are the leading cause of morbidity and mortality worldwide, accounting for 74% of deaths annually. Satellite imagery provides previously unattainable data about factors related to NCDs that overcome limitations of traditional, non-satellite-derived environmental data, such as subjectivity and requirements of a smaller geographic area of focus. This systematic literature review determined how satellite imagery has been used to address the top NCDs in the world, including cardiovascular diseases, cancers, chronic respiratory diseases, and diabetes.

**Methods:**

A literature search was performed using PubMed (including MEDLINE), CINAHL, Web of Science, Science Direct, Green FILE, and Engineering Village for articles published through June 6, 2023. Quantitative, qualitative, and mixed-methods peer-reviewed studies about satellite imagery in the context of the top NCDs (cancer, cardiovascular disease, chronic respiratory disease, and diabetes) were included. Articles were assessed for quality using the criteria from the Oxford Centre for Evidence-Based Medicine.

**Results:**

A total of 43 studies were included, including 5 prospective comparative cohort trials, 22 retrospective cohort studies, and 16 cross-sectional studies. Country economies of the included studies were 72% high-income, 16% upper-middle-income, 9% lower-middle-income, and 0% low-income. One study was global. 93% of the studies found an association between the satellite data and NCD outcome(s). A variety of methods were used to extract satellite data, with the main methods being using publicly available algorithms (79.1%), preprocessing techniques (34.9%), external resource tools (30.2%) and publicly available models (13.9%). All four NCD types examined appeared in at least 20% of the studies.

**Conclusion:**

Researchers have demonstrated they can successfully use satellite imagery data to investigate the world’s top NCDs. However, given the rapid increase in satellite technology and artificial intelligence, much of satellite imagery used to address NCDs remains largely untapped. In particular, with most existing studies focusing on high-income countries, future research should use satellite data, to overcome limitations of traditional data, from lower-income countries which have a greater burden of morbidity and mortality from NCDs. Furthermore, creating and refining effective methods to extract and process satellite data may facilitate satellite data’s use among scientists studying NCDs worldwide.

**Supplementary Information:**

The online version contains supplementary material available at 10.1186/s12889-024-20316-z.

## Introduction

Noncommunicable diseases (NCDs) account for 74% of global deaths annually, with cardiovascular diseases, cancers, chronic respiratory diseases, and diabetes responsible for over 80% of premature NCD mortalities [1]. NCDs are not limited to older adults, with 17 million deaths before age 70, predominantly in low- and middle-income countries [1]. In the United States, direct health costs related to NCDs exceed $1 trillion annually [2]. Unhealthy behaviors like smoking, poor diet, and physical inactivity increase NCD susceptibility [3]. The third largest underlying risk factor of chronic disease (after high blood pressure and tobacco usage) is air pollution, an environmental risk factor most often increasing the risk for three of the top four NCDs - cardiovascular disease, cancers, and chronic respiratory diseases [4].

Achieving the World Health Organization Sustainable Development Goal Target 3.4 of reducing premature NCD mortality by one-third by 2030 [5] is challenging, with most countries making minimal progress [6]. Identifying geographic locations with populations most at risk for NCDs is one step toward directing prevention-related policies and programs to achieve this goal [7]. Satellite technologies offer tools to help identify at-risk geographic locations that overcome limitations of traditional, non-satellite-derived environmental data (e.g., surveys and ground monitoring stations) such as subjectivity and being limited to smaller geographic areas. Satellite data is open source, available on a global scale, and has four resolutions: temporal, spatial, radiometric and spectral [8]. With over 400 Earth observation satellites orbiting our planet [9], satellite imagery data, often coupled with artificial intelligence (AI), has shown great promise in advancing areas of research outside of healthcare, such as earth science [10] and economics [11], and within health sciences, particularly in infectious disease [12–14]. While satellite data helps mitigate the problem of traditional environmental data availability, it presents new challenges in understanding what satellite data to use and how to interpret the data. Fortunately, publicly available algorithms, tools, and tutorials exist to help scientists extract, process, and interpret satellite data. Satellite imagery has been less commonly used for analyzing and managing NCDs [15].

Traditional (non-satellite-derived) environmental measurements have been successfully used in research in the form of surveys (e.g., light at night (LAN), greenspace) and ground monitoring stations (e.g., air pollution), both of which have limitations [8]. Survey data can be subjective and non-uniform, while ground monitoring station data is limited to areas within a close proximity to a station, most often a developed urban area [8]. Satellite data has been found to overcome these limitations by its being open source, available on a global scale, and having four resolutions: temporal, spatial, radiometric and spectral [8].

Satellite data is derived from remote sensors located on satellites. The amount of energy reflected, absorbed, or transmitted by any item on Earth creates a “spectral fingerprint.” Remote sensors can detect a number (specific to the type of remote sensor and called its spectral resolution) of spectral bands, which allows items to be identified by their spectral fingerprint [16, 17]. There are two types of sensors: passive sensors (e.g., radiometers and spectrometers operating in the visible, infrared, thermal infrared and microwave electromagnetic spectrum) that measure land and sea physical attributes (e.g., temperature, vegetation properties, aerosol properties, cloud properties) and active sensors (e.g., radar sensors, altimeters operating in the microwave band of the electromagnetic spectrum) that measure vertical profiles of land and sea attributes (e.g., forest structure, ice, aerosols). Satellites have specific orbits and sensor designs that dictate resolution [16]. How well a remote sensor can distinguish between small differences in energy is called its radiometric resolution, which is the amount of information in each pixel (e.g., 8 bit resolution that can store up to 256 values). Higher resolution means more detail, though this also requires more processing power. Spatial resolution is defined as the size of each pixel. For example, to see buildings you would need 10 m (m) spatial resolution, which represents a 10 m by 10 m square on the ground. Neighborhoods need 20 m spatial resolution, which represents a 20 m by 20 m square on the ground, while regional needs 1 km (km), which represents 1 km by 1 km square on the ground (national: 10 m, continent: 30 km and global: 110 km) [16]. Spectral resolution is defined by both the number of bands and how narrow the bands are. For example, 3–10 bands is referred to as multispectral, whereas hundreds or thousands of bans are hyperspectral. Temporal resolution is defined as the time it takes the satellite to complete one iteration of its orbit, which is dependent on its orbit, its swath, width and the specific sensor (e.g., Moderate Resolution Imaging Spectroradiometer (MODIS) on NASA’s Terra and Aqua satellite’s temporal resolution is 1–2 days) [16].

We aimed to provide the first systematic literature review focusing on how environmental factors data collected from satellite imagery has been used to examine risk, incidence, prevalence, or mortality related to an NCD, both methodologically and technically. This review can illuminate resources and methods for using emerging satellite imagery technologies to capture and analyze comprehensive data that can inform NCD prevention and control interventions and policies. By integrating satellite-derived data with ground-based monitoring systems, scientists and policymakers can better understand the risk and distribution of NCDs, allocate resources more effectively, and implement targeted strategies to lessen NCD burden.

## Methods

This systematic review was registered at PROSPERO (CRD42023433472). We followed the Preferred Reporting Items for Systematic Reviews and Meta-analyses (PRISMA) reporting guidelines [18].

### Data sources

We conducted a systematic review of literature related to satellite imagery and the top 4 NCDs (cardiovascular diseases, cancers, chronic respiratory diseases, and diabetes) in the world through June 6, 2023. We did not restrict our search to any start date. To gather relevant studies, we searched PubMed (including MEDLINE), CINAHL, Web of Science, Science Direct, Green FILE, and Engineering Village databases. See Additional file 1 for keyword search strings.

### Study selection

First, we removed duplicate studies. Next, at least two study authors independently assessed the remaining abstracts based on predetermined inclusion criteria of needing to examine the top four NCDs in the world using satellite imagery. We considered all quantitative, qualitative, and mixed method study designs written in English. Then 2 study authors independently evaluated the full-text articles for inclusion, with discrepancies resolved through discussion. Studies that were not about one or more NCDs, not about satellite imagery, or review articles were excluded. Another way to describe our inclusion criteria is using the PECO (Population, Exposure, Comparator, Outcomes) framework recommended for exploring associations of environmental and other exposures with health outcomes [19]. Table 1 presents the inclusion and exclusion criteria.

**Table 1:** Inclusion and exclusion criteria

	Inclusion criteria	Exclusion criteria
Population	Study population had at least one of the top four NCDs in the world (cardiovascular diseases, cancers, chronic respiratory diseases, or diabetes)	Study population did *not* have at least one of the top four NCDs in the world (cardiovascular diseases, cancers, chronic respiratory diseases, or diabetes)
Exposure	Any type of NCD risk factor(s)	None
Comparator	Patients prior to having NCD(s) or patients without NCD(s)	No comparator
Outcomes	Risk, incidence, prevalence, or mortality	No NCD-related outcome
Study design	Quantitative, qualitative, and mixed methods	Reviews
Methods	Used satellite imagery to examine the risk, incidence, prevalence, or mortality related to at least one of the top four NCDs in the world	Did *not* use satellite imagery to examine the risk, incidence, prevalence, or mortality related to at least one of the top four NCDs in the world
Language	English	Not available in English

### Data extraction

At least two study authors independently extracted information for each study that met the inclusion criteria, including the study aim, disease, geographic level, year of data collection, methods, tools and resources, data extracted from images, measures, results, and findings. The authors discussed and resolved any discrepancies in the extracted data. We assessed the quality of the evidence for each study using the criteria from the Oxford Centre for Evidence-Based Medicine [20]. The quality of each study was independently graded by two study authors, with any discrepancies resolved through discussion. Following is a description of the quality ratings: 1 for properly powered randomized clinical trials, 2 for well-designed controlled trials without randomization and prospective comparative cohort trials, 3 for case-control studies and retrospective cohort studies, 4 for case series with or without intervention and cross-sectional studies, and 5 for case reports or opinions of respected authorities.

We conducted a qualitative synthesis of satellite data by determining if an association (statistically significant relationship) was found between the satellite data and each study’s dependent variable (e.g., NCD outcome) and explored the authors’ statements about the value of using satellite data. We also recorded statements that included wording about satellite data such as “overcame the problem,” “great tool,” and “enhanced.” We used a spreadsheet so that at least two authors could track these associations and statements and used codes to categorize aspects about the value of satellite imagery for examining NCD outcomes for each article. Our analysis also included all authors reviewing the frequency of the findings and the wording of the statements. Based on this analysis, the authors developed themes about the value of satellite imagery for examining NCDs.

## Results

**Fig. 1:**
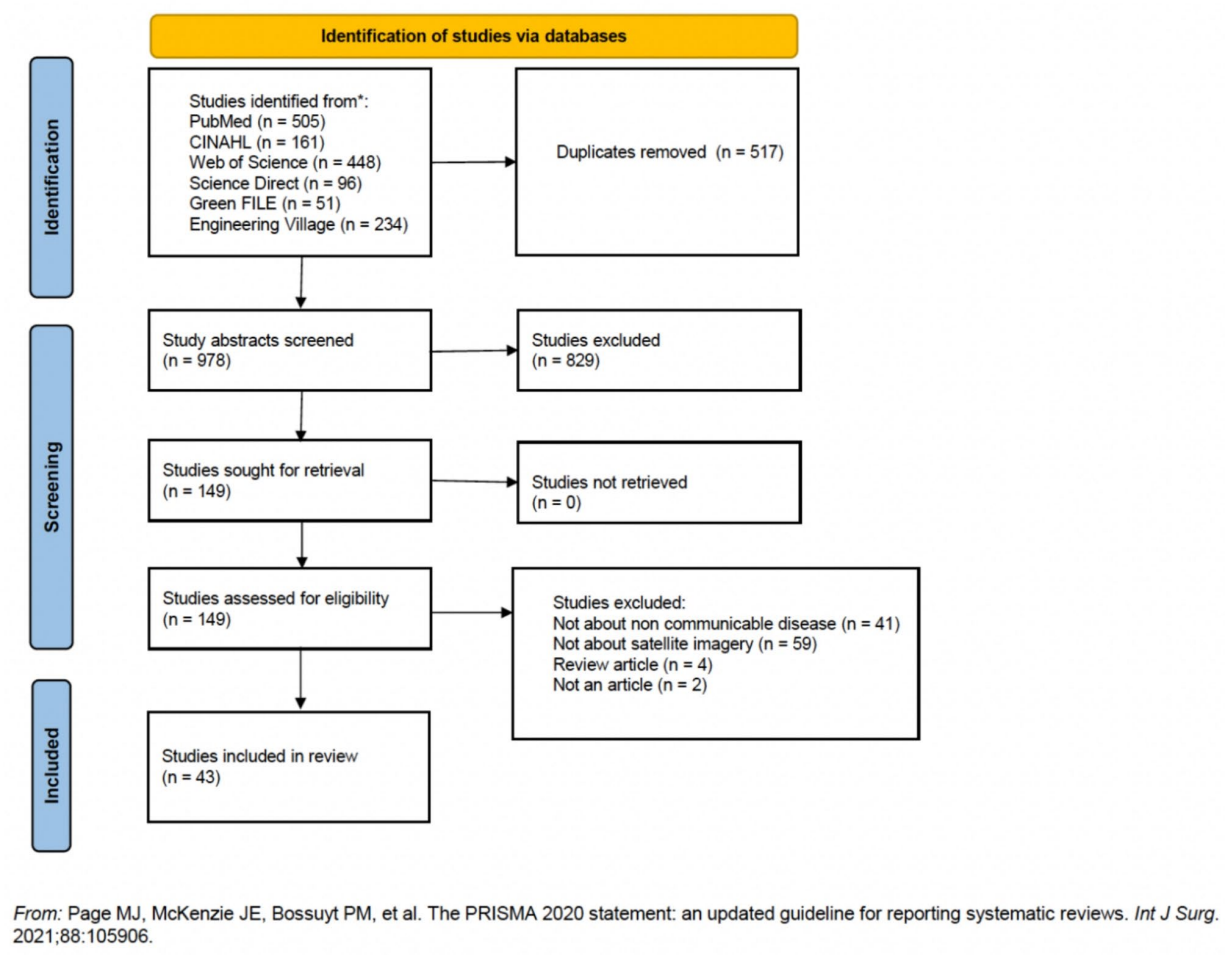
PRISMA flow diagram

We identified 1,495 articles from our database searches. After applying inclusion and exclusion criteria, 43 studies were selected for inclusion and 1,452 were excluded (Fig. 1). Table 2 includes details, quality assessment, and study authors’ statements about satellite value for all the reviewed studies and Table 3 presents the study characteristics, analysis, and data synthesis.

**Table 2:** Study details, quality, and authors’ statements about satellite value in reviewed articles

First Author (Year)	Geographic Level	Country Economies	Location	Disease(s) Addressed	Disease Outcome(s) Examined	Satellite Image Sources	Environmental Factors Extracted from Satellite Images	Data Extraction Methods	Study Quality Score	Relationship	Satellite Value Stated by Study Authors
Allen (2013) [35]	City	Lower Middle	Ulaanbaatar, Mongolia	Cancer (lung); Chronic respiratory disease	Mortality	Instrument: ETM + Landsat satellite	Air Pollution	Algorithm: Tassel cap transformation to simplify bands into three dimensions	4	Yes, greenness correlated with NO2 and SO2;	Overcomes problem of lack of spatial data satellite-based ETM + data, which have global coverage and are freely available
Bauer (2013) [46]	Metropolitan	High	Georgia, USA	Cancer (breast)	Incidence	Monitoring Program: DMSP; Nighttime DMSP-OLS	LAN	Algorithm; and External resource tool: Jenk’s Natural Break Method; ArcGIS	3	Yes, ground level circadian light and satellite photometers are significantly correlated;	Value found with overestimation that will ultimately attenuate results due to non-differential exposure misclassification
Bibault (2020) [57]	Census tract	High	Chicago, Dallas, Houston, Los Angeles, Phoenix, San Diego & San Jose, USA	Cancer	Prevalence	Application: Google Static Map zoom 18	Extracted numerical features	CNN: feature extractor, t-SNE	4	Yes, satellite features highly correlated with socioeconomic and health measures linked to cancer prevalence;	Value from accurately estimate cancer prevalence at high spatial resolution without use of surveys in areas of a few thousand people
Brown (2016) [61]	Census block	High	Miami, Florida, USA	Diabetes (obesity); Cardiovascular disease (hypertension)	Prevalence	Instrument - ASTER 15 × 15 m resolution - Terra satellite	Greenspace	Algorithm(s): comparing NDVIs (mean) with 0.1-unit change to NDVI (for land changes)	3	Yes, higher greenness is associated with better health	No specific mention of value of satellite data
Chan (2008) [21]	Metropolitan	High	Taipei, Taiwan	Cardiovascular disease (ischemic heart diseases); Chronic respiratory disease (COPD)	Incidence	Instrument: MODIS - Terra and Aqua satellites	Air pollution	Not stated	4	Yes, Asian dust storms are associated with an increased number of emergency visits for cardiopulmonary diseases if PM10 concentrations are above 90 mg/m3 during the storm-, the emergency visits for cardiovascular diseases, ischemic heart diseases, cerebrovascular diseases, and COPD during the Asian dust events are increased compared to the pre-dust period	Allowed confirmation of 85 dust storms
Dagliati (2016) [36]	County	High	Pavia area, Italy	Diabetes (type 2)	Prevalence	Satellite: Landsat L8 images (35 used)	Air pollution	Algorithm(s): Polynomial function from fitting process	3	Yes, spatial-temporal correlations h1bA1c and air pollution which follow the same trend	Ability to jointly study heterogeneous data (e.g., health care data and air pollution data extracted from satellites)
De Roos (2022) [22]	City	High	Philadelphia, USA	Chronic respiratory disease (asthma)	Prevalence	Instrument: MODIS- Terra satellite 25 m resolution	Greenspace	Algorithm(s): seasonal median NDVI value, 250 m buffer	3	Yes, correlated when each treatment case was looked at individually but no consistent association with overall greenness	Satellite data enhanced the data available and its usage
Eldeirawi (2019) [37]	City	High	Chicago, USA	Chronic respiratory disease (asthma)	Prevalence	Instrument: TM mission Landsat 5 satellite 30mx30m resolution	Greenspace	Algorithm(s): NDVI mean for multiple buffers; single day NDVI	4	Yes, increased protective effect of NDVI for children living with a smoker	No specific mention of value of satellite data
Evans (2013) [23]	Global	Global	Not applicable	Cancer (lung); Cardiovascular disease (ischemic heart disease); Chronic respiratory disease	Mortality	Instrument: MODIS and MISR - Terra satellite	Air pollution	Preprocessing: averaged PM2.5 levels from MODIS and MISR over 6 consecutive years	4	Yes, global fraction of adult mortality can be attributed to the anthropogenic component of PM2.5 for cardiopulmonary disease, lung cancer, and ischemic heart disease	More accurate than fixed site ground level measures alone and more similar to global chemical transport model simulations of anthropogenic PM2.5; Overcame limitation of only having data where ground monitors were available by using satellite data
Fan (2020) [38]	Household	Upper Middle	China	Chronic respiratory disease (COPD)	Prevalence	Instrument: ETM + mapper and OLI - Landsat satellite 30 m resolution	Greenspace; Air pollution	Algorithm(s), and preprocessing: NDVI (5 year-annual mean; negative index = 0); AOD with GWR; corrections: geometrical, atmospheric & color balance	4	Yes, higher level of NDVI increase trend of COPD prevalence	Ability to test exposure-outcome relationship using different NDVI buffer sizes and groupings
Gao (2022) [24]	Region	High	Salton Sea area, California	Chronic respiratory disease (asthma)	Prevalence	Instrument: MODIS (NDVI) - terra satellite; Satellite: Landsat 8 (water)	Greenspace; Water	Algorithm(s), external resource tool, and preprocessing: NDVI; water detect; Google gsutil; outlier; dummy;	3	Yes, used the spatial temporal change of NDVI to predict PM2.5 and PM10 concentration	Ability to enhance satellite data by incorporating more satellites in the future
Gariepy (2015) [62]	Region	High	Quebec, Canada	Diabetes (type 2)	Prevalence	Satellite imagery	Greenspace	Algorithm(s): NDVI (transformed into deciles)	3	Yes, higher level of greenness reduced risk of depression	No mention of satellite data value
Garzon-Chavez (2018) [25]	County (parish)	Upper Middle	Ecuador	Cancer (eye disease: Pterygium, cataract)	Incidence	Instrument: MODIS-Aqua satellite and OMI-Aura satellite	Air pollution	Algorithm(s): spectral ultraviolet algorithms (weighed for the erythemal effective action spectrum of human skin)	3	Yes, higher incidence rate correlated with area of highest up exposure which are at a low elevation	The observations by MODIS of consistent and steady presence of cloud highlights the influence of climate, especially for wet tropics, as a factor to reduce the extreme influence of equatorial latitude and surface elevation on UV irradiance which contribute to the cumulative exposure
Hidalgo-Garcia (2023) [53]	City	High	Granada, Spain	Cancer (stomach, colorectal, lung, prostate, and bladder)	Mortality	Satellite: Sentinel 3 and Sentinel 5P	Air pollution, Temperature, Greenspace	Algorithm(s), preprocessing, and external resource tool: LST, NDVI, NDBI and PV; AOD; QGIS & SNAP, reclassification, atmospheric correction	3	Yes positive correlation with an excess risk of developing stomach, colorectal, lung, prostate, and bladder cancer dementia, cerebrovascular disease, liver disease and suicidal	Satellite data can be extrapolated to other studies given the open-source satellite data and it’s use for determining LST and SUHI
Higgs (2015) [26]	Metropolitan	High	Athens, Greece	Chronic respiratory disease (asthma)	Prevalence	Instrument: MODIS - Terra and Aqua satellite	Air pollution	Algorithm(s), preprocessing, and external resource tool: Geophysical and aerosol retrieval algorithms, image selection criteria, MCMC, ArcMap	4	Yes, for individual variables AOD, NO, rH, temperature and an inverse association for ozone it found a small yet significant association with asthma hospital admission, did not find association when using multi-variable analysis	Demonstrates the ability to apply remote sensing data in the evaluation of health outcomes; The alignment process for remote sensing data is feasible; Missing data, can be reliably imputed to develop complete datasets
James (2016) [27]	Census tract	High	USA	Cancer; Diabetes; Cardiovascular disease (stroke); Chronic respiratory disease	Mortality	Instrument: MODIS - Terra satellite	Greenspace	Algorithm(s): contemporaneous NDVI for current season, cumulative NDVI (long term seasonal value and address changes)	2	Yes, higher levels of green vegetation were associated with decreased mortality	No mention of satellite data value
Jimenez (2020) [39]	Region	High	Eastern Massachusetts, USA	Diabetes (insulin resistance)	Risk	Satellite: Landsat 30 m resolution	Greenspace	Algorithm(s): NDVI (area weighted average July of follow-up)	3	No, did not find an association	NDVI is the most widely used satellite-derived indicator of green vegetation and allowed for a longitudinal exposure measure to green space using a residential address over the course of 12 years
Kim (2021) [60]	City	High	Korea	Chronic respiratory disease (COPD, post-bronchodilator response)	Prevalence	Satellite imagery	Air pollution	Algorithm(s) and preprocessing: AOD calibrated with ground monitoring data, land use regression over time	3	Yes, correlation between long term exposure to PM2.5 and emphysematous change in patients with normal lung function	No mention of satellite data value
Klompmaker (2022) [40]	Zip-code level	High	USA	Cardiovascular disease; Chronic respiratory disease	Prevalence	Satellite: Landsat 5, 7, and 8	Greenspace; Blue space	Algorithm(s), external resource tool, and preprocessing: mean summer NDVI (negative = 0); GEE	3	Yes, NDVI was weakly negatively correlated with percent park coverage, increase in NDVI was negatively correlated with CVD but not RSD hospitalizations in urban zip codes increase in NDVI was positively associated with RSD hospitalizations. For Medicare eligible and those living in low socioeconomic status neighborhoods in urban settings there was a negative association with CVD and RSD hospitalizations and percent park cover	No mention of satellite data value
Kloog (2008) [47]	City	High	Israel	Cancer (breast)	Incidence	Monitoring Program: DMSP	LAN	Algorithm and External resource tool: Jenk’s Natural Break Method; ArcGIS	3	Yes, a strong association between LAN and breast cancer	No mention of satellite data value
Lambert (2020) [41]	City	High	Sydney, Australia	Chronic respiratory disease (asthma; allergy)	Prevalence	Satellite: Landsat 7 Surface reflectance images 30 m x 30 m pixel resolution	Greenspace	Algorithm(s) and preprocessing: NDVI, cloud-free images from 2008 correlate w/season of lung measurement,	3	No, pollen count (from ground traps) was not found to be associated with NDVI	No mention of satellite data value
Liu (2023) [56]	City	Upper Middle	Qingdao, China	Diabetes; Cardiovascular disease (hypertension)	Prevalence	Satellite: Sentinel 2 10 m resolution	Greenspace	Algorithm(s) and external resource tool: NDVI (removed negative values); SAVI; VCF; EVI; ArcGIS	4	Yes, moderate correlation between NDVI and street greenspace quality, weak correlation between proximity to park-based greenspace and NDVI and street greenspace quality indicator and negative correlation between NDVI and prevalence of hypertension	No mention of satellite data value
Maharana (2018) [58]	Census tract	High	Los Angeles, Memphis, San Antonio, & Seattle, USA	Diabetes; Cardiovascular disease; and Cancers (risk factor: obesity)	Prevalence	Google Static Maps API	Extracted numerical features	CNN: VGG-CNN-F with Elastic net regression; cross validation	4	Yes, identified features of environment that have been associated with obesity from satellite images	Algorithms that use satellite data will lower cost and allow for investigating “place” when researching obesity prevalence
Medgyesi (2023) [48]	Census tract	High	California, Florisa, Louisiana, North Carolina, New Jersey, Pennsylvania, Atlanta Georgia, Detroit, & Michigan, USA	Cancer (endometrial)	Prevalence	Monitoring Program: DMSP, 1 km	LAN	Preprocessing: Transformed into units of radiance	2	No, did not find an association with LAN	No mention of satellite data value
Nguyen (2021) [28]	County (Australian Statistical Area Level 4)	High	New South Wales, Australia	Cardiovascular disease; Chronic respiratory disease	Incidence; mortality	Instruments: MODIS - Aqua and Terra satellite; CALIOP - CALIPSO satellite	Air pollution; Greenspace	WRF-Chem model (for hot spots) and external resource tool: MERRA-2	3	Yes, risk ratio of daily PM2.5 exposure and outpatient visits were higher on same day as exposure for chronic lower respiratory disease and cerebrovascular disease but not for ischemic heart disease	Allows for spatial variability unavailable from ground monitoring data. More accurate health estimate from exposure in urban, rural, and remote locations
Park (2022) [49]	State	High	California, Florisa, Louisiana, North Carolina, New Jersey, Pennsylvania, Atlanta Georgia, Detroit, & Michigan, USA	Cancer (liver)	Prevalence	Monitoring Program: DMSP	LAN	Preprocessing: Transformed into units of radiance	2	No, correlation with LAN found	No mention of satellite data value
Portnov (2016) [50]	Census tract	High	Connecticut, USA	Cancer (breast)	Incidence	Monitoring Program: DMSP	LAN	Preprocessing: radiance-calibrated image (average daily readings and remove cloud cover)	3	Yes, significant association with LAN	No mention of satellite data value
Prabhu (2019) [29]	City	Lower Middle	Dehradun city, India	Cancer	Risk	Instrument: MODIS - Terra satellite	Air pollution	External resource tool: Giovanni tool (NASA)	3	Yes, they found seasonal differences associated with air pollution and cancer risk	No mention of satellite data value
Prud’homme (2013) [30]	Census tract	High	Canada	Chronic respiratory disease (asthma, chronic bronchitis, allergy)	Prevalence	Instruments: MODIS; MISR - Terra satellite	Air pollution	GEOS-Chem model and preprocessing: AOD re-gridded to spatial resolution 10kmx10km	4	Yes, long term exposure to air pollution based on satellite data was associated with an increase of prevalence for allergies, asthma, current asthma and bronchitis	Not limited by proximity to monitoring station
Qazi (2019) [42]	City	Lower Middle	Isalamabad, Pakistan	Chronic respiratory disease (asthma, allergy)	Prevalence	Satellite: Landsat 7 30 m and SPOT-5 2.5 m	Greenspace (coverage of paper mulberry plant)	Supervised classification model, by location of cluster, plot mean values, temporal-spatial distribution	3	Yes, more severe levels of pollen allergy correlated to increase density of mulberry plant	Enables observing specific species of plant over time
Qu (2020) [31]	Region	Upper Middle	Guangdong Province, China	Diabetes (gestational diabetes mellitus)	Incidence	Instrument: MODIS 250 m NASA satellite	Greenspace	Algorithm(s) and spatial statistical model: NDVI multiple buffers	3	Yes, risk of GDM decreases monotonically with greater NDVI	No mention of satellite data value
Ramesh (2022) [32]	Zip-code Tabulation Area (for tropical storm Imelda, in Texas)	High	USA	Chronic respiratory disease (asthma)	Prevalence	Instrument: MODIS -NASA satellite; Satellite: Sentinel 1 A satellite	Flood	Algorithm(s): AER FloodScan	4	Yes, found increased flood was associated with increase ER visits for asthma	Allows for near-real time data
Silveira (2018) [43]	City	Upper Middle	Rio de Janeiro, Brazil	Cardiovascular disease (ischemic heart disease, cerebrovascular disease)	Mortality	Instrument: TM - Landsat 4–5 satellite, 30 m resolution	Greenspace	Algorithm(s) and preprocessing: NDVI (restrict to < 10% cloud cover), average NDVI & average NDVI by census sector	3	Yes, mortality rates for ischemic heart and cerebrovascular diseases are inversely associated with exposure to greenspace (when controlling socioeconomic status and air pollution) and found protective effect of greenspace is increased for lower socioeconomic levels	No mention of satellite data value
Stowell (2019) [33]	State	High	Colorado, USA	Cardiovascular disease; Chronic respiratory disease (bronchitis, asthma)	Prevalence	Instrument: MODIS - Terra and Aqua satellite	Air pollution	Algorithm(s): MAIAC: separating background ambient level from smoke level	3	Yes, respiratory disease outcomes increased in hospitalizations/ER visits, largest association was found with asthma	Allows for better defined local exposure for each event
Upegui (2012) [55]	Census tract	High	Besancon, France	Cancer (breast)	Incidence	Satellite: GeoEye	Greenspace	Algorithm(s) and external resource tool: GramSchmidt algorithm PCA and ISODATA for classification, ArcGIS	4	Yes, found association between cancer incidence and features	Found reliable population and disease rate estimate with Geo-Eye based classification; Overcomes cost limitations
Vargas-Cuentas (2018) [44]	County (province)	Lower Middle	Bolivia	Cardiovascular disease (Chagas disease)	Incidence	Instrument: OLI and TIRS -Landsat 8 satellite	Greenspace, Land Surface Temperature (LST);	Algorithm(s) and preprocessing: NDVI; NDWI; NDSI; tasseled cap index; relative humidity; correction: radiometric, geometric, & atmospheric	4	Yes, incidence of Chagas cases is linked to certain parameters such as: temperature, the water stress of vegetation, water bodies and accumulated precipitation	Can determine environment in which disease transmission, distribution, and evolution occur over time
Walker (2022) [45]	City	High	Vancouver, Canada	Diabetes	Prevalence	Satellite: Landsat 5	Greenspace	Algorithm(s): NDVI to get four separate metrics (median, standard deviation, 95th percentile and 5th percentile)	3	Yes, diabetes and greenspace are associated	No mention of satellite data value
Wang (2019) [63]	County	High	Miami, Florida	Cardiovascular disease (myocardial infarction, ischemic heart disease, heart failure, atrial fibrillation)	Prevalence	Instrument: ASTER 15 × 15-meter spatial resolution	Greenspace	Algorithm(s): NDVI (mean at block level categorized into low, middle and high)	4	Yes, higher greenspace is associated with decrease in odds of three types of cardiovascular related chronic conditions	Allows for replication in any neighborhood in the United States
Xiao (2020) [51]	State; City	High	USA	Cancer (breast)	Incidence	Monitoring Program: DMSP	LAN	External resource tool: nighttime radiance value by linking geocoded address at baseline to high-dynamic LAN; ArcGIS	2	Yes, increase LAN exposure increases risk of breast cancer	Overcomes subjectiveness of surveys on LAN
Xiao (2021) [52]	State	High	USA	Cancer (pancreatic)	Incidence	Monitoring Program: DMSP	LAN	External resource tool, and preprocessing: transformed to units of radiance; ArcGIS	2	Yes, higher LAN exposure was associated with increased pancreatic cancer risk	No mention of satellite data value
Yitshak (2015) [64]	Census tract	High	China	Cardiovascular disease (stroke)	Incidence	Daily satellite remote sensing 1 km- NASA satellite	Air pollution	Algorithm(s): MAIMC	4	Yes, higher risk of ischemic stroke associated with daily average PM2.5 and PM10 concentration on day of event for young subjects and suggests stronger effect of traffic pollution and not pollution of natural sources	Ability to include populations not located near remote monitoring station; As technology evolves, higher resolution data will allow for more precision matching daily exposure to home and work address
Yuan (2023) [54]	Country	Upper-Middle	China	Cancer (brain)	Prevalence; Incidence; Mortality	Instrument: OMI - NASA Aura satellite; Monitoring Program: China Environmental Monitoring Center satellite	Air pollution	Global CTM model; radiation transfer model; DOAS inversion technique	3	Yes, increased formaldehyde is associated with increased indoor formaldehyde	Satellite data allows for better spatial coverage; Easier to obtain than -in situ formaldehyde pollution data
Zhang (2019) [34]	City; Provincial	Upper-Middle	Henan, Hubei, Anhui, Jiangsu, & Shandong province, China	Cardiovascular disease; Chronic respiratory disease	Mortality	Instrument: MODIS - Terra and Aqua satellites	Air pollution	Preprocessed: adaptive bias correction method	4	Yes, air pollution had a greater impact on cardiovascular disease than respiratory disease	Provides reliable ground-level concentrations of air pollution

Abbreviations: AER, Atmospheric and Environmental Research; AOD, Aerosol Optical Depth; AOL, Airspace Operations Laboratory; ASTER, Advanced Spaceborne Thermal Emissions and Reflection Radiometer; CALIOP, Cloud-Aerosol; CNN, Convolution Neural Network; COPD, Chronic Obstructive Pulmonary disease; CTM, chemical transport model; CVD, Cardiovascular disease; DMSP, Defense Meteorological Satellite Program; DMSP-OLS, Defense Meteorological Satellite Program - Operational Linescan System; DOAS, Differential Optical Absorption Spectroscopy, EOS, Earth Observation System; ER, emergency room; ETM+, Enhanced Thematic Mapper Plus; EVI, Enhanced Vegetation Index; LAN, Light at Night; GDM, gestational diabetes mellitus; GEE, Google Earth Engine; GEOS-chem, Goddard Earth Observing System Chemical transport model; GWR, Geographically Weighted Regression; LST, Land Surface Temperature; m, Meter; MCMC, Markov Chain Monte Carlo; MAIAC, Multi-angle Implementation of Atmospheric Correction; MERRA-2, Modern-Era Retrospective Analysis for Research and Applications, Version 2; MISR, Multi-angle Imaging SpectroRadiometer; MODIS, Moderate Resolution Imaging Spectroradiometer; NASA, National Aeronautics and Space Administration; NDBI, Normalized Difference Built-up Index; NDSI, Normalized Difference Snow Index; NDVI, Normalized Difference Vegetation Index; NDWI, Normalized Difference Water Index; OLI, Operational Land Imager; OMI, Ozone Monitoring Instrument; PCA, Principal Component Analysis; Pm2.5, Particulate matter in air with diameter less than 2.5 micrometers; Pm10, Particulate matter in air with diameter less than 10 micrometers; PV, Photosynthetic Vegetation; RSD, Chronic respiratory disease, SAVI, Soil Adjusted Vegetation Index; SNAP, Sentinel Application Platform; SUHI, Surface urban heat island; TIRS, Thermal Infrared Sensor; t-SNE, t-distributed Stochastic Neighbor Embedding; TM, Thematic Mapper; VCF, Vegetation Continuous Fields; WRF-chem, Weather Research and Forecast model coupled with chemistry

**Table 3:** Characteristics of the studies, analysis, and data synthesis (*n* = 43)

	Number of studies
**Study quality score**	
1 (Randomized controlled trial)	0 (%)
2 (Prospective comparative cohort trial)	5 (11.6%)
3 (Case-control studies; retrospective cohort study)	22 (51.2%)
4 (Cross-sectional study)	16 (37.2%)
5 (Case reports)	0 (0.0%)
**Country economies** ^**a**^	
High-income	31 (72.1%)
Upper-middle-income	7 (16.2%)
Lower-middle-income	4 (9.3%)
Low-income	0 (0.0%)
**Geographic level**	
Global	1 (2.3%)
Country	1 (2.3%)
Region	4 (9.1%)
State/Province	3 (6.8%)
County	5 (11.6%)
Metropolitan area	3 (6.8%)
City	14 (31.8%)
Zip code	2 (4.5%)
Census tract or census block	9 (20.9%)
Household	1 (2.3%)
**Disease outcomes examined** ^**b**^	
Prevalence	23 (53.5%)
Incidence	13 (30.2%)
Mortality	8 (18.6%)
Risk	2 (4.7%)
**Environmental factors extracted from satellite images** ^**b, c**^	
Greenspace^d^	20 (46.5%)
Air pollution (including aerosol optical depth; surface reflectance, cloud fraction, PM2.5 level, and smoke level)	16 (37.2%)
Light at night (LAN)	7 (15.9%)
Flooding	1 (2.3%)
Temperature	2 (4.6%)
**Satellite image source(s)** ^**b, c**^	
Satellite: Aura, Aqua or Terra:	
Instrument: Moderate Resolution Imaging Spectroradiometer (MODIS) Instrument: Multi-Angle Imaging Spectroradiometer (MISR) Instrument: Advanced Spaceborne Thermal Emissions and Reflection Radiometer (ASTER) (*using Terra satellite*) Instrument: Ozone Monitoring Instrument (OMI) (*using Aqua satellite*)	13 (29.5%) 3 (6.8%) 2 (4.5%) 2 (4.5%)
Satellite: LandSat − 4, 5, 7, or 8:	
Instrument: Enhanced Thematic (ETM) Instrument: Operational Land Imager (OLI) Instrument: Thermal Infrared Sensor (TIRS) (*using landsat 8 satellite*) Instrument: Thematic (TM) (*using landsat 4 or 5 satellite)* Instrument: unspecified	2 (4.5%) 2 (4.5%) 1 (2.3%) 2 (4.5%) 7 (16.3%)
Monitoring Program: U.S. Defense Meteorological Satellite Program (DMSP)	9 (20.5%)
Satellite: Sentinel 2, 3, or 5P	3 (6.8%)
Application: Google Static Map	2 (4.5%)
Satellite: CALIPSO	1 (2.3%)
Satellite: China Environmental Monitoring Center Satellite	1 (2.3%)
Satellite: GeoEye	1 (2.3%)
Satellite: SPOT-5	1 (2.3%)
**Satellite data extraction method(s)** ^**a**^	
**Algorithm** AER FloodScan (32) AOD (general, with GWR or with land use regression) (38,53,60) Geophysical and aerosol retrieval (26) GrahamSchmidt (55)	**34 (79.1%)** 1 3 1 1
Index:	
NDVI (13,24,27,31,37–41,43–45,56,61–63) Tassel cap (45,63) EVI, NDWI, NDSI, SAVI, or VCF (35,44)	6 2 2
Jenk’s Natural Break Method (46,47) MAIAC (33,64) Polynomial function (36) Sentinel algorithms (index: LST, NDVI, NDBI and PV) (53) Spectral Ultraviolet (25) Water detect (24)	2 2 1 1 1 1
**CNN** CNN with t-SNE (57) VGG-CNN-F (58)	**2 (4.7%)** 1 1
**External resources tool** ArcGIS (46,47,51,52,55,56) ArcMap (26) GEE (40) Google gsutil tool (24) MERRA-2 (28) NASA Giovanni (29) QGIS (53) SNAP (53)	**13 (30.2%)** 6 1 1 1 1 1 1 1
**Image preprocessing** General (23,26,30,34,38,40,41,43,44,48,49,51–53) Calibration (50,60)	**16 (37.2%)** 14 2
**Model**	**6 (14%)**
Global transport:	
WRF-Chem (28) GEOS-Chem (30) Unspecified (54) Radiation transfer (54) Spatial-statistical (31) Supervised classification (42)	1 1 1 1 1 1
**Not stated** (21)	**1 (2.3%)**

Abbreviations: AER, Atmospheric and Environmental Research; AOD, Aerosol Optical Depth; CTM, chemical transport model; EVI, Enhanced Vegetation Index; GEE, Google Earth Engine; GEOS-chem, Goddard Earth Observing System Chemical transport model; GWR, Geographically Weighted Regression; LST, Land surface temperature; MAIAC, Multi-angle Implementation of Atmospheric Correction; MERRA-2, Modern-Era Retrospective Analysis for Research and Applications, Version 2; NASA, National Aeronautics and Space Administration; NDBI, Normalized Difference Built-up Index; NDSI, Normalized Difference Snow Index; NDVI, Normalized Difference Vegetation Index; NDWI, Normalized Difference Water Index; PV, Photosynthetic Vegetation; SAVI, Soil Adjusted Vegetation Index; SNAP, Sentinel Application Platform; t-SNE, t-distributed Stochastic Neighbor Embedding; VCF, Vegetation Continuous Fields; WRF-chem, Weather Research and Forecast model coupled with chemistry

^a^ One study is not included here because it was global [23]

^**b**^ These totals do not add up to 43 because some studies fit into multiple categories

^c^ Two articles extracted numerical features using artificial intelligence (AI) [57, 58]

^d^ Two articles that include greenspace also includes blue space, or water [24, 40]

The study publication dates spanned over 15 years, 2008–2023, with more than half published within the 5 years before our study search end date. Overall, 70% of the studies were from high-income countries, with over half of those from the United States. The remaining studies were from middle-income countries, and none were from low-income countries. The majority of studies (66%) used satellite data examined at the city, census tract or census block, or county level. About half of the study designs were retrospective cohort and about one-third were cross-sectional. The 12% of studies that fit in the most rigorous study design category for this review (had prospective study designs) all focused on cancer. There were no randomized control trials or case reports. Regarding disease outcomes examined, prevalence was an outcome in over half of the studies (53.5%), incidence in about 30% of the studies, mortality in just under 20%, and disease risk in just under 5% (Table 2).

30% of the studies used satellite images from MODIS [21–34], an instrument located on NASA’s Aura, Terra, and Aqua satellites, and 16% used unspecified instruments located on NASA’s Landsat satellites [24, 35–45]. The other major satellite image source was the United States Space Force’s Department of Meteorological Satellite Program (DMSP) [46–52], with images used in 21% of the studies. MODIS and Landsat were primarily used to extract air pollution and greenspace data, while DMSP was primarily used to detect LAN. Greenspace was the most frequently extracted data, with nearly half of the studies examining this feature. The next most frequently extracted data was air pollution, appearing in 37.2% of the studies (Table 3).

**Fig. 2:**
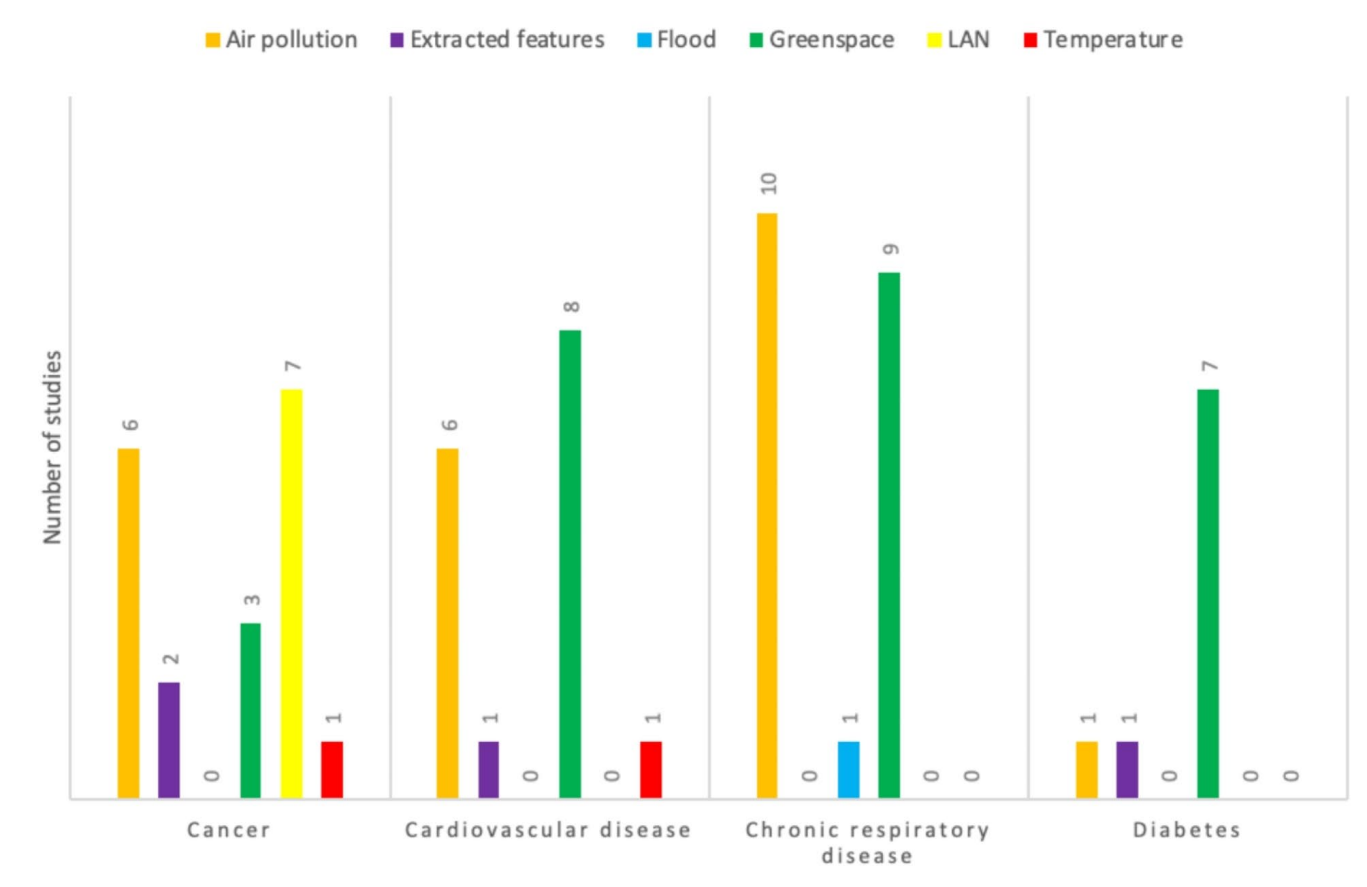
The number of studies of each type of satellite data for each noncommunicable disease. “LAN” is “light at night” and refers to ambient light exposure at night. “Extracted features” refers to features extracted using machine learning to inform a machine learning model. “Air pollution” refers to aerosol optic depth measures, particle matter in air with diameter less than 2.5 micrometers, particle matter in air with diameter less than 10 micrometers, “Flood” refers to changes in land and water surface due to rainfall. “Greenspace” refers to normalized difference vegetation index (NDVI), enhanced vegetation index (EVI), photosynthetic vegetation (PV), and soil adjusted vegetation index (SAVI). “Temperature” refers to the land surface temperature (LST) and surface urban heat island (SUHI)

All 4 major NCDs examined—cardiovascular disease, cancers, chronic respiratory disease, and diabetes—appeared in at least 20% of the studies, with chronic respiratory diseases and cancers each appearing in about 40%. Cardiovascular disease, chronic respiratory disease, and diabetes studies heavily used greenspace and air pollution data. All studies using LAN examined cancer outcomes [46–52], with 4 of the 7 specific to breast cancer [46, 47, 50, 51]. DMSP data was from 1996 to 97 and was used as a baseline for determining LAN in 6 [47–52] of the 7 articles [46–52]. Approximately one-third of the cancer studies included air pollution data [23, 25, 29, 35, 53, 54], while less than one-fifth included greenspace data [27, 53, 55] (Table 3). Air pollution was the primary data extracted for chronic respiratory disease, closely followed by greenspace. We found the reverse with the studies on cardiovascular disease; greenspace was the primary data extracted, closely followed by air pollution. Greenspace was the primary data extracted for diabetes studies (Fig. 2).

The majority of the articles (60%) used data from earlier than 5 years from the publication date and matched health data year(s) to satellite data year(s). Of the 10 articles [26, 37, 41, 45, 47–52] using data from earlier than 10 years from the publication date, 6 used data from DMSP [47–52], and 3 used data from LandSat [37, 41, 45]. All but 3 articles [39, 48, 49] in the review found associations between the satellite data and NCD outcomes of risk, incidence, prevalence, or mortality. For example, Fan and colleagues [38] found a positive association between neighborhood greenness and COPD prevalence using the NDVI based on satellite imagery. Two of the studies that found no association used LAN and cancer [48, 49]; however, both found that LAN was a valid representation of circadian rhythm disruption. The third article found no association between greenspace in early life and insulin resistance in adolescence [39] (Table 2).

All data extracted in the studies in this review were related to a previously known disease risk factor. That is, no studies introduced a new disease risk factor that had not been established in prior research. Additionally, the majority of the studies included covariates such as sociodemographic factors like age and income level or health-related factors like body mass index (BMI) and smoking. Most studies focused on one type of satellite data, such as greenspace. However, 9% of the studies [22, 24, 28, 37, 38, 40, 44, 53, 56] examined multiple environmental factors extracted from satellites, such as greenspace and air pollution, in studying the incidence [28, 44], prevalence [22, 24, 37, 38, 40, 56], and mortality [28, 53] of NCDs. Two articles used a type of AI, convolutional neural networks (CNN), to extract numerical features from satellite imaging. One article [57] used a CNN with t-SNE (t-distributed Stochastic Neighbor Embedding) to verify the capacity of the neural network to extract relevant features related to cancer prevalence. The other article [58] used a visual geometry group fast convolution neural network (VGG-CNN-F), a network previously described by Chatfield and colleagues [59] with elastic net regression to prevent overfitting of the model to training data and minimize mean cross-validation error in a study examining obesity prevalence.

All articles used satellite data extracted based on geolocation(s) specific to the population of interest’s location (versus using data from a convenience sample based on data availability) using one or two of a variety of methods (Table 3). Existing publicly available algorithms were the primary method for satellite data extraction, with such algorithms used for analyzing 85% of greenspace data (primarily the normalized difference vegetation index, or NDVI) and 50% of air pollution data (such as the multi-angle Implementation of Atmospheric Correction (MAIAC) and deriving aerosol optical depth (AOD) with geographically weighted regression (GWR), primarily for particulate matter 2.5, or PM2.5). Two articles used the Jenk’s Natural Break method algorithm to classify LAN data [46, 47]. Image preprocessing methods performed on raw satellite image data to prepare it for further data processing were referenced in just under half of the studies. Examples of image preprocessing are LAN data transformed into radiance [49, 50, 52] or determining image inclusion according to criteria such as 10% or less cloud cover. Image preprocessing calibration, gauging the data with a standard scale, was used in two articles [50, 60]. One article calibrated LAN with satellite sensor data to provide average daily radiance [50] and another calibrated satellite air pollution data with ground data using a land-use regression model [60]. One-quarter of the studies referenced external resource tools that extract data from satellite images, such as ArcGIS, MERRA-2, NASA’s Giovanni tool, and the Sentinel Application Platform [26, 28, 29, 46, 47, 51–53, 56]. Five articles used models, three of which were specific to chemistry: WRF-Chem [28], GEOS-chem [30], and an unspecified global chemical transport model [54]. Yuan and colleagues [54] also used the radiation transfer model and the differential absorption spectroscopy inversion technique. Qu and colleagues [31] used a spatial-statistical model with the NDVI to derive an estimate of residential greenness, and Qazi and colleagues [42] used a model for supervised classification. The “satellite data extraction method(s)” section in Table 3 shows the breakdown of satellite data extraction methods used in the studies.

The vast majority (90%) of studies found an association between their dependent variable and the satellite-derived data [21–38, 40, 42–47, 50–53, 55–58, 60–65] (Table 2). For example, both Bauer and colleagues [46] and Kloog and colleagues [47] found breast cancer incidence was associated with high LAN exposure. Fan and colleagues [38] found a significant positive association between COPD prevalence and greenness (NDVI). Another 2% of studies [39] found value in satellite data but no association with their dependent variable [39]. In this article, Jimenez and colleagues [39] found that NDVI is the most widely used satellite-derived indicator of green space and can be used as a longitudinal exposure measure. Furthermore, 58% of the articles stated that satellite data overcomes the difficulties present in research when geographic areas do not have environmental data or the available ground data is sparse [21–26, 28, 30, 32–36, 38, 39, 42, 44, 51, 53, 55, 57, 58, 63–65]. For example, Prud’homme [30] (2013) and Yitshak [64] (2015) specifically noted the limitation of ground data for air pollution, proximity to monitoring stations, and sparse spatial data, which can be overcome by using satellite data. The final two columns of Table 2 shows if the dependent variable and satellite-derived data were related and any claims stated by the study authors about the value of using satellite data. We found two themes regarding the value of using satellite imagery to examine risk, incidence, prevalence, and mortality related to NCDs. The first theme was that satellite data overcomes problems of sparse or missing spatial and temporal data. Traditional environmental data (non-satellite data) is limited by the range of each sensor and the completeness of data. Satellite data complements traditional data and extends the availability of environmental spatial and temporal data to a global scale. The following representative quote illustrates such sentiments:

In particular, we have clearly shown that, thanks to data availability and big data technologies, it is now possible to jointly study heterogenous data, such as health care and air pollution information extracted from satellites. This provides an unprecedented opportunity to improve our understanding of phenomena by extracting unseen temporal and spatial correlations [36].

Use of satellite images has become a great tool for epidemiology because with this technological advance we can determine the environment in which transmission occurs, the distribution of the disease and its evolution over time [44].

The second theme was the ability of open-source satellite data to enable studies to be extrapolated to other areas. Traditional data sources are specific to a distinct geographic area, whereas satellite data often allows for (spatial and temporal) data to be available globally. The following representative quote illustrates such sentiments:

The results of studies like ours could be extrapolated to other cities, given that the use of Sentinel 3 satellite images lies within the reach of the entire scientific community, and their use for determining land surface temperature (LST) and surface urban heat island (SUHI) is straight forward [53].

Our findings will have important public health implications for policy makers when they are planning the size, shape, density and accessibility of surrounding green spaces in living areas [38].

## Discussion

Satellite imagery has been used in a variety of ways to address NCDs. A key finding of this review is that nearly all studies found an association between at least one type of satellite data and one NCD. Most of the studies used existing publicly available algorithms to extract data from images. Furthermore, a couple of studies [57, 58] attempted to harness the power of AI to see beyond predetermined features.

Over half of the studies analyzed satellite data based on geolocation(s) specific to the research population of interest’s location at the city, census tract, or census block level. One reason the authors may have chosen these geographic levels could be because sociodemographic and health-related data, types of data that have been integral to NCD studies [3], is often available at these levels [3, 66]. Furthermore, by adding satellite data at these geographic levels, study authors may have been able to fill gaps in environmental factors when using ground data alone, such as in Allen and colleagues’ [35] examination of air pollution in the city of Ulaanbaatar, Mongolia. Just one article used satellite data globally; it replicated previous studies that either excluded areas without ground monitoring or were limited by “coarse spatial resolution” [23].

Over 80% of premature deaths caused by NCD occur in lower-income countries [1], yet just one-quarter of the studies in our review were from lower-income countries. Satellites orbiting the Earth capture data from across the globe, including areas without robust healthcare data collection, such as lower-income countries [8]. Using satellite data for these countries could better enable disease surveillance, tracking health trends and risk factors, and informed healthcare decision-making [15, 67]. One potential strategy for using satellite data in lower-income countries is identifying trends between certain satellite-derived data and health outcomes in similar countries or regions that have health data available and extrapolating those trends to the lower-income country of focus. A similar method has been succeesfully used with satellite imagery data in the field of economics [68]. However, a limiting factor for using satellite imagery for health is a lack of proper tools, knowledge, and skills in collecting and analyzing satellite data among researchers, engineers, and government employees [69]. This is particularly problematic in lower-income countries with fewer educational resources to train people [69, 70]. One potential solution to address the lack of training is to use the publicly available governmental and university-based tutorial programs and resources designed to make satellite data easier to use [69–72]. Additional file 2 presents resources to help researchers, scientists, and policymakers understand, find, and use satellite imagery data.

Most reviewed articles discussed how satellite data is an asset to NCD research by providing open-access environmental data that surmounts the constraints of ground-based data collection methods and availability. However, it is important that investigators consider tradeoffs between levels of spatial, temporal, and spectral resolution when choosing their satellite remote sensing data sources [15]. Researchers can use guidance from organizations such as NASA [73, 74] and the European Space Agency [75, 76] to help determine the optimal scale of data needed for their research. For example, MODIS (on Terra, Aura, Aqua, and Sentinel 1a satellites) is an instrument that produces moderate-resolution images, while Sentinel 2 and 3 instruments produce high-resolution images. The choice between using MODIS or Sentinel 2 or Sentinel 3 would depend on the investigator team’s resources (e.g., computing processing power and available investigator hours) and the data needs (e.g., general greenspace in a city versus specific greenspace by city block) for their research questions.

While satellite imagery has existed for over a half-century [77], not until recently did scientists have a method to process the vast amounts of data amassed by observing our planet from space [78, 79]. AI can quickly and efficiently extract meaningful patterns, trends, and insights from satellite images. Most articles in this review (95%) examined previously known environmental factors (e.g., air pollution, LAN) using satellite image data. The paucity of studies employing AI to analyze satellite image data for NCD research–just two studies in this review–is notable given that other fields (e.g., waste management [80, 81], agriculture [82, 83], urban planning [82, 84], and defense [82, 85]) have more readily adopted AI methods to process satellite image data. Within health sciences, satellite imagery coupled with AI has been used to study patterns in infectious diseases [12, 13, 71]. With 241 in-orbit Earth observation satellites registered with the United Nations and that number growing [86], there is an opportunity to expand the use of AI and satellite data to monitor and analyze NCD risk for informing policy and programmatic decisions to improve noncommunicable disease outcomes.

Several studies used existing algorithms to measure satellite data. These algorithms allowed the researchers to avoid developing a new measure and better enabled comparisons between studies. We recommend that researchers continue testing these existing algorithms and develop and test new algorithms to measure satellite data to help facilitate future satellite imagery-based NCD research. Such algorithms could be constructed by researchers or generated using AI and validated through research studies. The existence of previously validated algorithms may help epidemiologists and other individuals focused on studying NCD conduct more robust satellite data-based studies and avoid the need to create and study the properties of a new measure.

Our study deviated from the original protocol in a few ways. First, due to the quantitative nature of the studies, we ended up using the assessment of quality from the Oxford Centre for Evidence-Based Medicine instead of the Mixed Methods Appraisal Tool. Second, we focused on the top four NCDs in the world because the World Health Organization highlights these four diseases as the deadliest diseases [1]. Third, we added more technical engineering and environmental databases to our literature search to ensure we captured as many articles as possible that fit our search criteria.

This review has some limitations. First, it was limited in scope to peer-reviewed literature; thus, it could be missing case reports and other grey literature contributions. Second, there is a risk of publication bias in a review of published studies. Third, due to the heterogeneity of the research methods across studies, we did not perform a meta-analysis to quantitatively examine how satellite imagery has been used to address the top NCDs in the world. This limits the depth of the analysis that could be achieved through more rigorous statistical exploration. Fourth, given that most studies are from developed regions, findings are skewed toward higher-income countries. Future studies should explore ways to include more diverse geographical inputs to research using satellite imagery in examining noncommunicable diseases.

## Conclusions

Overall, this systematic review found satellite data to be an asset to NCD research. However, given the recent proliferation of satellites and the emerging capabilities of AI, using satellite imagery data to address the global health threat of NCDs has barely scratched the surface, particularly for locations most vulnerable to NCDs, such as low- and middle-income countries. Scientists and policymakers worldwide should take concerted and collaborative action to keep pace with the advancement of satellite imagery to produce better data-driven health outcomes.

## Electronic supplementary material

Below is the link to the electronic supplementary material.

Supplementary Material 1

Supplementary Material 2

## Data Availability

Data is provided within the manuscript or supplementary information files.

## Abbreviations

AI: Artificial intelligence
AOD: Aerosol Optical Depth
BMI: Body Mass Index
CNN: Convolutional Neural Networks
DMSP: Department of Meteorological Satellite Program
GWR: Geographically weighted regression
KM: Kilometer
LAN: Light at night
M: Meter
MAIAC: Multi-angle Implementation of Atmospheric Correction
MODIS: Moderate Resolution Imaging Spectroradiometer
NASA: National Aeronautics and Space Administration
NCD: Noncommunicable disease
NDVI: Normalized Difference Vegetation Index
PM2.5: Particulate matter 2.5
PRISMA: Preferred Reporting Items for Systematic Reviews and Meta-analyses
t-SNE: t-distributed Stochastic Neighbor Embedding

## References

[1] World Health Organization. Noncommunicable Diseases. 2022. https://www.who.int/news-room/fact-sheets/detail/noncommunicable-diseases

[2] Waters H, Graf M. The Costs of Chronic Disease in the U.S.. Milken Institute; 2018 Aug [cited 2023 Sep 20]. https://milkeninstitute.org/sites/default/files/reports-pdf/ChronicDiseases-HighRes-FINAL_2.pdf

[3] Centers for Disease Control and Prevention. Centers for Disease Control and Prevention. 2023 [cited 2023 Aug 26]. PLACES: Local Data for Better Health. https://www.cdc.gov/places/index.html

[4] World health Organization. Environmental risk factors and NCDs. [cited 2024 Mar 21]. https://www.who.int/teams/noncommunicable-diseases/integrated-support/environmental-risk-factors-and-ncds

[5] World Health Organization W health statistics. The Global Health Observatory. 2023. WHO Indicator Data: Global Health Observatory. https://www.who.int/data/gho/data/themes/topics/indicator-groups/indicator-group-details/GHO/sdg-target-3.4-noncommunicable-diseases-and-mental-health#:~:text=Indicator Groups-,SDG Target 3.4 %7C Noncommunicable diseases and mental health%3A By 2030,mental health and well%2Dbeing

[6] Watkins DA, Msemburi WT, Pickersgill SJ, Kawakatsu Y, Gheorghe A, Dain K, et al. NCD countdown 2030: efficient pathways and strategic investments to accelerate progress towards the sustainable development goal target 3.4 in low-income and middle-income countries. Lancet. 2022;399(10331):1266–78. 35339227 10.1016/S0140-6736(21)02347-3 PMC8947779

[7] World Health Organization. Global NCD Compact 2020–2030. [cited 2023 Aug 9]. https://www.who.int/initiatives/global-noncommunicable-diseases-compact-2020-2030/achievements

[8] NASA Earth Science Data Systems. What is Remote Sensing? | Earthdata. 2019 [cited 2024 Mar 21]. https://www.earthdata.nasa.gov/learn/backgrounders/remote-sensing

[9] World Economic Forum. 2020 [cited 2023 Aug 29]. Who owns our orbit: Just how many satellites are there in space? https://www.weforum.org/agenda/2020/10/visualizing-easrth-satellites-sapce-spacex/

[10] Barnard PL, Vitousek S. Earth science looks to outer space. Nat Geosci. 2023;16(2):108–9.

[11] Yeh C, Perez A, Driscoll A, Azzari G, Tang Z, Lobell D, et al. Using publicly available satellite imagery and deep learning to understand economic well-being in Africa. Nat Commun. 2020;11(1):2583. 32444658 10.1038/s41467-020-16185-w PMC7244551

[12] Amoroso N, Cilli R, Maggipinto T, Monaco A, Tangaro S, Bellotti R. Satellite data and machine learning reveal a significant correlation between NO2 and COVID-19 mortality. Environ Res. 2022;204:111970. 34474031 10.1016/j.envres.2021.111970 PMC8403556

[13] Desai AN, Kraemer MU, Bhatia S, Cori A, Nouvellet P, Herringer M, et al. Real-time epidemic forecasting: challenges and opportunities. Health Secur. 2019;17(4):268–75. 31433279 10.1089/hs.2019.0022 PMC6708259

[14] Rogers DJ, Randolph SE, Snow RW, Hay SI. Satellite imagery in the study and forecast of malaria. Nature. 2002;415(6872):710–5. 11832960 10.1038/415710a PMC3160466

[15] Jia P, Stein A, James P, Brownson RC, Wu T, Xiao Q, et al. Earth Observation: investigating Noncommunicable diseases from Space. Annu Rev Public Health. 2019;40(1):85–104. 30633713 10.1146/annurev-publhealth-040218-043807

[16] Earthdata | Earthdata. [cited 2022 Nov 8]. https://www.earthdata.nasa.gov/

[17] ESA - Eduspace EN - Home. - What is remote sensing?. 2010 [cited 2024 Jan 25]. https://www.esa.int/SPECIALS/Eduspace_EN/SEMF9R3Z2OF_0.html#:~:text=Remote sensing is a way of collecting and,data being in direct contact with the object

[18] Page MJ, McKenzie JE, Bossuyt PM, Boutron I, Hoffmann TC, Mulrow CD, et al. The PRISMA 2020 statement: an updated guideline for reporting systematic reviews. Int J Surg. 2021;88:105906. 33789826 10.1016/j.ijsu.2021.105906

[19] Morgan RL, Whaley P, Thayer KA, Schünemann HJ. Identifying the PECO: a framework for formulating good questions to explore the association of environmental and other exposures with health outcomes. Environ Int. 2018;121(Pt 1):1027. 30166065 10.1016/j.envint.2018.07.015 PMC6908441

[20] Howick J, Chalmers I, Glasziou P, Greenhalgh T, Heneghan C, Liberati A et al. OCEBM Levels of Evidence. Centre for Evidence-Based Medicine (CEBM), University of Oxford. 2021.

[21] Chan CC, Chuang KJ, Chen WJ, Chang WT, Lee CT, Peng CM. Increasing cardiopulmonary emergency visits by long-range transported Asian dust storms in Taiwan. Environ Res. 2008;106(3):393–400. 17959168 10.1016/j.envres.2007.09.006

[22] De Roos AJ, Kenyon CC, Yen YT, Moore K, Melly S, Hubbard RA, et al. Does living near Trees and other Vegetation affect the contemporaneous odds of Asthma Exacerbation among Pediatric Asthma patients? J Urban Health. 2022;99(3):533–48. 35467328 10.1007/s11524-022-00633-7 PMC9187838

[23] Evans J, Van Donkelaar A, Martin RV, Burnett R, Rainham DG, Birkett NJ, et al. Estimates of global mortality attributable to particulate air pollution using satellite imagery. Environ Res. 2013;120:33–42. 22959329 10.1016/j.envres.2012.08.005

[24] Gao J, Liu J, Xu R, Pandey S, Vankayala Siva VSKS, Yu D. Environmental Pollution Analysis and Impact Study—A Case Study for the Salton Sea in California. Atmosphere. 2022;13(6):914.

[25] Garzon-Chavez DR, Quentin E, Harrison SL, Parisi AV, Butler HJ, Downs NJ. The geospatial relationship of pterygium and senile cataract with ambient solar ultraviolet in tropical Ecuador. Photochem Photobiol Sci. 2018;17(8):1075–83. 29926886 10.1039/c8pp00023a

[26] Higgs G, Sterling DA, Aryal S, Vemulapalli A, Priftis KN, Sifakis NI. Aerosol Optical Depth as a measure of Particulate exposure using Imputed Censored Data, and relationship with Childhood Asthma Hospital Admissions for 2004 in Athens, Greece. Environ Health Insights. 2015;9s1:EHI.S15665. 10.4137/EHI.S15665 PMC4412425 25987842

[27] James P, Hart JE, Banay RF, Laden F. Exposure to greenness and mortality in a nationwide prospective cohort study of women. Environ Health Perspect. 2016;124(9):1344–52. 27074702 10.1289/ehp.1510363 PMC5010419

[28] Nguyen HD, Azzi M, White S, Salter D, Trieu T, Morgan G, et al. The summer 2019–2020 wildfires in East Coast Australia and their impacts on Air Quality and Health in New South Wales, Australia. Int J Environ Res Public Health. 2021;18(7):3538. 33805472 10.3390/ijerph18073538 PMC8038035

[29] Prabhu V, Shridhar V, Choudhary A. Investigation of the source, morphology, and trace elements associated with atmospheric PM10 and human health risks due to inhalation of carcinogenic elements at Dehradun, an Indo-Himalayan city. SN Appl Sci. 2019;1(5):429.

[30] Prud’homme G, Dobbin NA, Sun L, Burnett RT, Martin RV, Davidson A, et al. Comparison of remote sensing and fixed-site monitoring approaches for examining air pollution and health in a national study population. Atmos Environ. 2013;80:161–71.

[31] Qu Y, Yang B, Lin S, Bloom MS, Nie Z, Ou Y, et al. Associations of greenness with gestational diabetes mellitus: the Guangdong Registry of congenital heart Disease (GRCHD) study. Environ Pollut. 2020;266:115127. 32650202 10.1016/j.envpol.2020.115127

[32] Ramesh B, Jagger MA, Zaitchik BF, Kolivras KN, Swarup S, Yang B, et al. Estimating changes in emergency department visits associated with floods caused by Tropical Storm Imelda using satellite observations and syndromic surveillance. Health Place. 2022;74:102757. 35131607 10.1016/j.healthplace.2022.102757

[33] Stowell JD, Geng G, Saikawa E, Chang HH, Fu J, Yang CE, et al. Associations of wildfire smoke PM2.5 exposure with cardiorespiratory events in Colorado 2011–2014. Environ Int. 2019;133:105151. 31520956 10.1016/j.envint.2019.105151 PMC8163094

[34] Zhang D, Bai K, Zhou Y, Shi R, Ren H. Estimating ground-level concentrations of Multiple Air Pollutants and their Health impacts in the Huaihe River Basin in China. Int J Environ Res Public Health. 2019;16(4):579. 30781540 10.3390/ijerph16040579 PMC6407116

[35] Allen RW, Gombojav E, Barkhasragchaa B, Byambaa T, Lkhasuren O, Amram O, et al. An assessment of air pollution and its attributable mortality in Ulaanbaatar, Mongolia. Air Qual Atmos Health. 2013;6(1):137–50. 23450113 10.1007/s11869-011-0154-3 PMC3578716

[36] Dagliati A, Marinoni A, Cerra C, Decata P, Chiovato L, Gamba P, et al. Integration of administrative, clinical, and Environmental Data to support the management of type 2 diabetes Mellitus: from satellites to Clinical Care. J Diabetes Sci Technol. 2016;10(1):19–26. 10.1177/1932296815619180 PMC4738227 26630915

[37] Eldeirawi K, Kunzweiler C, Zenk S, Finn P, Nyenhuis S, Rosenberg N, et al. Associations of urban greenness with asthma and respiratory symptoms in Mexican American children. Ann Allergy Asthma Immunol. 2019;122(3):289–95. 30557617 10.1016/j.anai.2018.12.009

[38] Fan J, Guo Y, Cao Z, Cong S, Wang N, Lin H, et al. Neighborhood greenness associated with chronic obstructive pulmonary disease: a nationwide cross-sectional study in China. Environ Int. 2020;144:106042. 32827808 10.1016/j.envint.2020.106042

[39] Jimenez MP, Oken E, Gold DR, Luttmann-Gibson H, Requia WJ, Rifas-Shiman SL, et al. Early life exposure to green space and insulin resistance: an assessment from infancy to early adolescence. Environ Int. 2020;142:105849. 32593049 10.1016/j.envint.2020.105849 PMC7784302

[40] Klompmaker JO, Laden F, Browning MHEM, Dominici F, Ogletree SS, Rigolon A, et al. Associations of parks, greenness, and blue space with cardiovascular and respiratory disease hospitalization in the US Medicare cohort. Environ Pollut. 2022;312:120046. 36049575 10.1016/j.envpol.2022.120046 PMC10236532

[41] Lambert KA, Lodge C, Lowe AJ, Prendergast LA, Thomas PS, Bennett CM, et al. Pollen exposure at birth and adolescent lung function, and modification by residential greenness. Allergy. 2019;74(10):1977–84. 30934123 10.1111/all.13803

[42] Qazi S, Iqbal J, Khan JA. Assessment of the health impact of paper mulberry (Broussonetia papyrifera L.), an invasive plant species in Islamabad, Pakistan. Geospatial Health. 2019 Nov 12 [cited 2023 Jul 12];14(2). https://geospatialhealth.net/index.php/gh/article/view/727 10.4081/gh.2019.727 31724384

[43] Silveira IHD, Junger WL. Green spaces and mortality due to cardiovascular diseases in the city of Rio De Janeiro. Rev Saúde Pública. 2018;52:49. 29723390 10.11606/S1518-8787.2018052000290 PMC5947462

[44] Vargas-Cuentas NI, Roman-Gonzalez A, Mantari AA, Muñoz LA. Chagas disease study using satellite image processing: a Bolivian case. Acta Astronaut. 2018;144:216–24.

[45] Walker BB, Brinkmann ST, Große T, Kremer D, Schuurman N, Hystad P, et al. Neighborhood Greenspace and Socioeconomic Risk are Associated with Diabetes Risk at the Sub-neighborhood Scale: results from the prospective Urban and Rural Epidemiology (PURE) Study. J Urban Health. 2022;99(3):506–18. 35556211 10.1007/s11524-022-00630-w PMC9187823

[46] Bauer SE, Wagner SE, Burch J, Bayakly R, Vena JE. A case-referent study: light at night and breast cancer risk in Georgia. Int J Health Geogr. 2013;12(1):23. 23594790 10.1186/1476-072X-12-23 PMC3651306

[47] Kloog I, Haim A, Stevens RG, Barchana M, Portnov BA. Light at night co-distributes with incident breast but not Lung Cancer in the Female Population of Israel. Chronobiol Int. 2008;25(1):65–81. 18293150 10.1080/07420520801921572

[48] Medgyesi DN, Trabert B, Fisher JA, Xiao Q, James P, White AJ, et al. Outdoor light at night and risk of endometrial cancer in the NIH-AARP diet and health study. Cancer Causes Control. 2023;34(2):181–7. 36222982 10.1007/s10552-022-01632-4 PMC10236480

[49] Park Y, Ramirez Y, Xiao Q, Liao LM, Jones GS, McGlynn KA. Outdoor light at night and risk of liver cancer in the NIH-AARP diet and health study. Cancer Causes Control. 2022;33(9):1215–8. 35840828 10.1007/s10552-022-01602-w PMC12895398

[50] Portnov BA, Stevens RG, Samociuk H, Wakefield D, Gregorio DI. Light at night and breast cancer incidence in Connecticut: an ecological study of age group effects. Sci Total Environ. 2016;572:1020–4. 27531467 10.1016/j.scitotenv.2016.08.006

[51] Xiao Q, James P, Breheny P, Jia P, Park Y, Zhang D, et al. Outdoor light at night and postmenopausal breast cancer risk in the NIH-AARP diet and health study. Int J Cancer. 2020;147(9):2363–72. 32488897 10.1002/ijc.33016

[52] Xiao Q, Jones RR, James P, Stolzenberg-Solomon RZ. Light at night and risk of pancreatic Cancer in the NIH-AARP Diet and Health Study. Cancer Res. 2021;81(6):1616–22. 33514513 10.1158/0008-5472.CAN-20-2256 PMC8693799

[53] Hidalgo-García D, Arco-Díaz J. Spatiotemporal analysis of the surface urban heat island (SUHI), air pollution and disease pattern: an applied study on the city of Granada (Spain). Environ Sci Pollut Res. 2023;30(20):57617–37. 10.1007/s11356-023-26564-7 PMC10163141 36971934

[54] Yuan Q, Shen H, Li T, Li Z, Li S, Jiang Y, et al. Deep learning in environmental remote sensing: achievements and challenges. Remote Sens Environ. 2020;241:111716.

[55] Upegui E, Viel JF. GeoEye Imagery and Lidar Technology for small-area Population Estimation: an epidemiological viewpoint. Photogramm Eng Remote Sens. 2012;78(7):693–702.

[56] Liu Y, Zhao B, Cheng Y, Zhao T, Zhang A, Cheng S, et al. Does the quality of street greenspace matter? Examining the associations between multiple greenspace exposures and chronic health conditions of urban residents in a rapidly urbanising Chinese city. Environ Res. 2023;222:115344. 36693460 10.1016/j.envres.2023.115344

[57] Bibault JE, Bassenne M, Ren H, Xing L. Deep learning prediction of Cancer Prevalence from Satellite Imagery. Cancers. 2020;12(12):3844. 33352801 10.3390/cancers12123844 PMC7766226

[58] Maharana A, Nsoesie EO. Use of Deep Learning to Examine the Association of the built Environment with Prevalence of Neighborhood adult obesity. JAMA Netw Open. 2018;1(4):e181535. 30646134 10.1001/jamanetworkopen.2018.1535 PMC6324519

[59] Chatfield K, Simonyan K, Vedaldi A, Zisserman A. Return of the Devil in the Details: Delving Deep into Convolutional Nets. arXiv; 2014 [cited 2023 Sep 14]. http://arxiv.org/abs/1405.3531

[60] Kim Y, Bak SH, Kwon SO, Kim H, Kim WJ, Lee CY. Association between Long-Term exposure to PM2.5 and lung imaging phenotype in CODA Cohort. Atmosphere. 2021;12(2):282.

[61] Brown SC, Lombard J, Wang K, Byrne MM, Toro M, Plater-Zyberk E, et al. Neighborhood Greenness and Chronic Health conditions in Medicare Beneficiaries. Am J Prev Med. 2016;51(1):78–89. 27061891 10.1016/j.amepre.2016.02.008

[62] Gariepy G, Kaufman JS, Blair A, Kestens Y, Schmitz N. Place and health in diabetes: the neighbourhood environment and risk of depression in adults with type 2 diabetes. Diabet Med. 2015;32(7):944–50. 25440062 10.1111/dme.12650

[63] Wang K, Lombard J, Rundek T, Dong C, Gutierrez CM, Byrne MM, et al. Relationship of Neighborhood Greenness to Heart Disease in 249 405 US Medicare Beneficiaries. J Am Heart Assoc. 2019;8(6):e010258. 30835593 10.1161/JAHA.118.010258 PMC6475064

[64] Yitshak Sade M, Novack V, Ifergane G, Horev A, Kloog I. Air Pollution and ischemic stroke among young adults. Stroke. 2015;46(12):3348–53. 26534971 10.1161/STROKEAHA.115.010992

[65] Yuan Y, Wu Y, Zhao H, Ren J, Su W, Kou Y, et al. Tropospheric formaldehyde levels infer ambient formaldehyde-induced brain diseases and global burden in China, 2013–2019. Sci Total Environ. 2023;883:163553. 37100142 10.1016/j.scitotenv.2023.163553

[66] Bureau UC. Census.gov. [cited 2023 Aug 26]. Census.gov. https://www.census.gov/en.html

[67] Schwalbe N, Wahl B. Artificial intelligence and the future of global health. Lancet. 2020;395(10236):1579–86. 32416782 10.1016/S0140-6736(20)30226-9 PMC7255280

[68] Lehnert P, Niederberger M, Backes-Gellner U, Bettinger E. T Jaworski editor 2023 Proxying economic activity with daytime satellite imagery: filling data gaps across time and space. PNAS Nexus 2 4 pgad099. 37077886 10.1093/pnasnexus/pgad099 PMC10108942

[69] Rolf E, Proctor J, Carleton T, Bolliger I, Shankar V, Ishihara M, et al. A generalizable and accessible approach to machine learning with global satellite imagery. Nat Commun. 2021;12(1):4392. 34285205 10.1038/s41467-021-24638-z PMC8292408

[70] Malekzadeh A, Michels K, Wolfman C, Anand N, Sturke R. Strengthening research capacity in LMICs to address the global NCD burden. Glob Health Action. 2020;13(1):1846904. 33373280 10.1080/16549716.2020.1846904 PMC7782223

[71] Canadian Space Agency, Canadian Space A. 2018 [cited 2023 Aug 11]. How satellites help you stay healthy. https://www.asc-csa.gc.ca/eng/satellites/everyday-lives/how-satellites-help-you-stay-healthy.asp

[72] mosaiks.org. 2023 [cited 2023 Aug 11]. mosaiks.org. https://www.mosaiks.org

[73] National Oceanic and Atmospheric Administration. GOES-R Algorithm Theoretical Basis Documents. 2023 [cited 2023 Dec 3]. https://www.star.nesdis.noaa.gov/goesr/documentation_ATBDs.php

[74] NASA. Worldview. [cited 2023 Sep 17]. https://worldview.earthdata.nasa.gov/

[75] Earth Online. [cited 2023 Sep 17]. https://earth.esa.int/eogateway/

[76] EUMETSAT. EUMETSAT | Monitoring the weather and climate from space | EUMETSAT. [cited 2023 Sep 17]. https://www.eumetsat.int/

[77] Gettelman A, Geer AJ, Forbes RM, Carmichael GR, Feingold G, Posselt DJ, et al. The future of Earth system prediction: advances in model-data fusion. Sci Adv. 2022;8(14):eabn3488. 35385304 10.1126/sciadv.abn3488 PMC8985915

[78] Chen CP, Zhang CY. Data-intensive applications, challenges, techniques and technologies: a survey on Big Data. Inf Sci. 2014;275:314–47.

[79] Kotawadekar R. 9 - Satellite data: big data extraction and analysis. In: Binu D, Rajakumar BR, editors. Artificial Intelligence in Data Mining. Academic Press; 2021 [cited 2023 Aug 26]. pp. 177–97. https://www.sciencedirect.com/science/article/pii/B9780128206010000082

[80] Abu Qdais H, Shatnawi N. Assessing and predicting landfill surface temperature using remote sensing and an artificial neural network. Int J Remote Sens. 2019;40(24):9556–71.

[81] Baghanam AH, Vakili AT, Nourani V, Dąbrowska D, Soltysiak M. AI-based ensemble modeling of landfill leakage employing a lysimeter, climatic data and transfer learning. J Hydrol. 2022;612:128243.

[82] Tahir A, Munawar HS, Akram J, Adil M, Ali S, Kouzani AZ, et al. Automatic target detection from Satellite Imagery using machine learning. Sensors. 2022;22(3):1147. 35161892 10.3390/s22031147 PMC8839603

[83] Tariq A, Siddiqui S, Sharifi A, Shah SHIA. Impact of spatio-temporal land surface temperature on cropping pattern and land use and land cover changes using satellite imagery, Hafizabad District, Punjab, Province of Pakistan. Arab J Geosci. 2022;15(11):1045.

[84] Jiang H, Peng M, Zhong Y, Xie H, Hao Z, Lin J, et al. A Survey on Deep Learning-based change detection from high-resolution remote sensing images. Remote Sens. 2022;14(7):1552.

[85] Bistron M, Piotrowski Z. Artificial Intelligence Applications in Military systems and their influence on sense of security of citizens. Electronics. 2021;10(7):871.

[86] United Nations Register of Objects. Launched into Outer Space. [cited 2023 Aug 29]. https://www.unoosa.org/oosa/en/spaceobjectregister/index.html

