# Supplementary material for: Examining noncommunicable diseases using satellite imagery: a systematic literature review
Source: BMC Public Health. 2024 Oct 10;24:2774. doi: 10.1186/s12889-024-20316-z (PMC11468461; doi:10.1186/s12889-024-20316-z)
Supplement: Supplementary file 1 — Supplementary Material 1 [file 12889_2024_20316_MOESM1_ESM.docx]

**Additional File 1**

Table: Search Strategy

| **Database** | **Search Strategy** |
| --- | --- |
| CINAHL Complete | 1. AB “noncommunicable disease*” OR AB "chronic disease" OR AB "chronic illness" OR AB cancer OR AB "cardiovascular disease*" OR AB "heart disease" OR  AB stroke OR AB "chronic respiratory disease*" OR AB "chronic obstructive pulmonary disease" OR AB "COPD" OR AB asthma OR AB "lung disease" OR AB "sleep apnea" OR AB "cystic fibrosis" OR AB "diabetes" OR AB "kidney disease"  OR TI “noncommunicable disease*” OR Ti "chronic disease" OR TI "chronic illness" OR TI cancer OR TI "cardiovascular disease*" OR TI "heart disease" OR  TI stroke OR TI "chronic respiratory disease*" OR TI "chronic obstructive pulmonary disease" OR TI "COPD" OR TI asthma OR TI "lung disease" OR TI "sleep apnea" OR TI "cystic fibrosis" OR TI "diabetes" OR TI "kidney disease"  2. AB "satellite imag*" OR AB "earth observation*" OR AB "satellite photo*" OR AB "Satellite picture*" OR AB "space picture*" OR AB "space photo*" OR AB "space image*" OR AB "aerial system*" OR AB "unmanned aerial vehicle* image*" OR AB "aerial image*" OR AB "geospatia*" OR AB "remote sensing data" OR TI "satellite imag*" OR TI "earth observation*" OR TI "satellite photo*" OR TI "Satellite picture*" OR TI "space picture*" OR TI "space photo*" OR TI "space image*" OR TI "aerial system*" OR TI "unmanned aerial vehicle* image*" OR TI "aerial image*" OR TI "geospatia*" OR TI "remote sensing data"  3. 1 and 2  Limiter: Through June 2023, English Language, Peer Reviewed |
| Engineering Village | 1. noncommunicable disease OR chronic disease OR chronic illness OR cancer OR cardiovascular disease OR heart disease OR stroke OR chronic respiratory disease OR chronic obstructive pulmonary disease OR COPD OR asthma OR lung disease OR sleep apnea OR cystic fibrosis OR diabetes OR kidney disease  2. satellite imag* OR satellite photo OR Satellite picture OR space picture OR aerial image  3. 1 AND 2  Limiters: 2000-2023, English, Journal Article |
| Green File | 1.  noncommunicable disease OR chronic disease OR chronic illness OR cancer OR cardiovascular disease OR heart disease OR stroke OR chronic respiratory disease OR chronic obstructive pulmonary disease OR COPD OR asthma OR lung disease OR sleep apnea OR cystic fibrosis OR diabetes OR kidney disease  2. satellite imag* OR earth observation OR Satellite photo* OR Satellite picture* OR remote sensing data  3. 1 AND 2  Limiter: Peer Reviewed, Academic Journals, Publication date 2005-2022 |
| PubMed | 1.((“noncommunicable disease*”[ Title/Abstract]) OR ("chronic disease"[Title/Abstract]) OR ("chronic illness"[Title/Abstract]) OR  ("cancer"[Title/Abstract]) OR ("cardiovascular disease*"[Title/Abstract]) OR ("heart disease"[Title/Abstract]) OR ("stroke"[Title/Abstract]) OR ("chronic respiratory disease*"[Title/Abstract]) OR ("chronic obstructive pulmonary disease"[Title/Abstract]) OR ("COPD"[Title/Abstract]) OR ("asthma"[Title/Abstract]) OR ("lung disease"[Title/Abstract]) OR ("sleep apnea"[Title/Abstract]) OR ("cystic fibrosis"[Title/Abstract]) OR ("diabetes"[Title/Abstract]) OR ("kidney disease"[Title/Abstract])) AND (("satellite imag*"[Title/Abstract]) OR ("earth observation*"[Title/Abstract]) OR ("satellite photo*"[Title/Abstract]) OR ("Satellite picture*"[Title/Abstract]) OR ("space picture*"[Title/Abstract]) OR ("space photo*"[Title/Abstract]) OR ("space image*") OR ("aerial system*"[Title/Abstract]) OR ("from space"[Title/Abstract]) OR ("unmanned aerial vehicle* image*"[Title/Abstract]) OR ("aerial image*"[Title/Abstract]) OR ("geospatia*"[Title/Abstract]) OR ("remote sensing data"[Title/Abstract]))  Limiters: from June 6, 2023, English, Exclude preprints |
| Science Direct | 1. ("satellite image" OR "earth observation" OR "satellite photo" OR “remote sensing”) AND ("chronic disease" OR "chronic illness" OR "noncommunicable disease")  2. ("satellite image" OR "earth observation" OR "satellite photo" OR “remote sensing”) AND ("cancer" OR "cardiovascular disease" OR "heart disease" OR “stroke”)  3. ("satellite image" OR "earth observation" OR "satellite photo" OR “remote sensing”) AND ("sleep apnea" OR "cystic fibrosis" OR "diabetes" OR "kidney disease" OR “asthma”) |
| Web of Science | 1. ((AB=( “noncommunicable disease” OR  "chronic disease" OR "chronic illness" OR  “cancer” OR  "cardiovascular disease" OR "heart disease" OR  stroke OR "chronic respiratory disease" OR "chronic obstructive pulmonary disease" OR "Chronic Obstructive Pulmonary Disease" OR “asthma” OR "lung disease" OR "sleep apnea" OR "cystic fibrosis" OR "diabetes" OR "kidney disease") OR TI=( “noncommunicable disease” OR  "chronic disease" OR "chronic illness" OR  “cancer” OR  "cardiovascular disease" OR "heart disease" OR  “stroke” OR "chronic respiratory disease" OR "chronic obstructive pulmonary disease" OR "Chronic Obstructive Pulmonary Disease" OR “asthma” OR "lung disease" OR "sleep apnea" OR "cystic fibrosis" OR "diabetes" OR "kidney disease"))) AND (AB=("satellite image" OR "earth observation*" OR "satellite photo" OR "Satellite picture" OR "space picture" OR "space photo" OR "space image" OR "aerial system*" OR "unmanned aerial vehicle image" OR "aerial image" OR "geospatial" OR "remote sensing data" OR “from space”) OR TI=("satellite image" OR "earth observation" OR "satellite photo" OR "Satellite picture" OR "space picture" OR "space photo" OR "space image" OR "aerial system" OR "unmanned aerial vehicle* image" OR "aerial image" OR "geospatial" OR "remote sensing data" OR “from space”))  Limiters: Article; Review; Early Access |

*In addition to the database searches, other potentially fitting articles the researchers identified while reviewing the retrieved articles were screened.
