# Supplementary material for: Examining noncommunicable diseases using satellite imagery: a systematic literature review
Source: BMC Public Health. 2024 Oct 10;24:2774. doi: 10.1186/s12889-024-20316-z (PMC11468461; doi:10.1186/s12889-024-20316-z)
Supplement: Supplementary file 2 — Supplementary Material 2 [file 12889_2024_20316_MOESM2_ESM.docx]

**Additional File 2.** Satellite imagery data resources

| **Resource name and its organization** | **Resource description** |
| --- | --- |
| United States  Multi-task Observation using SAtellite Imagery & Kitchen Sinks (MOSAIKS) - *University of California, Santa Barbara & University of California, Berkeley* (1)  Algorithm Theoretical Basis Document – *NASA* (2)  Earth Observing System Data and Information System (EOSDIS) – *NASA* (3)  Europe  European Operational Satellite Agency for Monitoring the Weather, Climate and the Environment from Space (EUMETSAT) – *ESA* (4)  Earth Online – *ESA* (5)  Copernicus Open Access Hub – *ESA* (6)  Science Toolbox Exploitation Platform (STEP) – *ESA* (7) | An easy-to-use tool designed to take satellite data from anywhere on earth, fill data gaps, and provide an actionable summary using satellite imagery and machine learning (SIML)  A list of algorithms with documentation providing details about the specific calculations of geophysical quantities  Federated architecture that allows access to individual components of NASA’s earth observation data, analysis tools, and visualization tools to enable discovery, data access, and distribution  Provides access to tools to download and view ESA’s satellite data, both historical and near real-time  Provides access to ESA’s earth observation news, information, tools, and data  Open access to Sentinel-1, 2, 3, and 5P data, analysis tools, visualization tools, and user guides  SNAP installers and Sentinel Toolbox, which provide a way to process and analyze earth observation data; Offers open-source common architecture for ESA |

Abbreviations: NASA, National Aeronautics and Space Administration; ESA, European Space Agency

References for Additional File 2:

1. mosaiks.org [Internet]. 2023 [cited 2023 Aug 11]. mosaiks.org. Available from: https://www.mosaiks.org

2. National Oceanic and Atmospheric Administration. GOES-R Algorithm Theoretical Basis Documents [Internet]. 2023 [cited 2023 Dec 3]. Available from: https://www.star.nesdis.noaa.gov/goesr/documentation_ATBDs.php

3. NASA. Worldview [Internet]. [cited 2023 Sep 17]. Available from: https://worldview.earthdata.nasa.gov/

4. EUMETSAT. EUMETSAT | Monitoring the weather and climate from space | EUMETSAT [Internet]. [cited 2023 Sep 17]. Available from: https://www.eumetsat.int/

5. Earth Online [Internet]. [cited 2023 Sep 17]. Available from: https://earth.esa.int/eogateway/

6. Open Access Hub [Internet]. [cited 2023 Sep 17]. Available from: https://scihub.copernicus.eu/

7. STEP – Science Toolbox Exploitation Platform [Internet]. 2023 [cited 2023 Sep 17]. Available from: https://step.esa.int/main/
